# Triggering final follicular maturation- hCG, GnRH-agonist or both, when and to whom?

**DOI:** 10.1186/s13048-015-0187-6

**Published:** 2015-08-21

**Authors:** Raoul Orvieto

**Affiliations:** Sackler Faculty of Medicine, Tel Aviv University, Tel Aviv, Israel; Department of Obstetrics and Gynecology, Infertility and IVF Unit, Sheba Medical Center, Ramat Gan, 52621 Israel

**Keywords:** hCG, GnRH agonist, Ovulation, Trigger, OHSS, Controlled ovarian hyperstimulation, Oocyte quality

## Abstract

Controlled ovarian hyperstimulation (COH) which combines GnRH antagonist co-treatment and GnRH-agonist (GnRHa) trigger has become a common tool aiming to *eliminate* severe early OHSS and to support the concept of an OHSS-free clinic. However, due to the reported significantly reduced clinical, efforts have been made to improve reproductive outcome. One of the suggested optional strategies aiming to improve outcome was the addition of low-dose (1500 IU) HCG bolus, administered, concomitant, 35 h or 5 days after the triggering bolus of GnRHa. All these regimens were demonstrated to rescue the luteal phase, resulting in improved reproductive outcome in patients at risk to develop severe OHSS, compared to GnRHa trigger alone, however, with the questionable ability to *eliminate* severe OHSS.

Moreover, following the observations demonstrating comparable or even better oocyte\embryos quality following GnRHa, compared to hCG trigger, and the different effects of LH and hCG on the downstream signaling of the LH receptor, GnRHa is now offered concomitant to the standard hCG trigger dose to improve oocyte/embryo yield and quality. GnRHa and hCG may be offered either concomitantly, 35–37 h prior to oocyte retrieval (dual trigger), or 40 h and 34 h prior to oocyte retrieval, respectively (double trigger).

## Introduction

Assisted reproduction technology (ART) practitioners seek a balance between optimum ovarian stimulation and successful treatment outcome with minimal rate of severe ovarian hyperstimulation syndrome (OHSS) or multiple pregnancies.

During IVF treatment, human chorionic gonadotropin (hCG) is usually used as a surrogate LH surge to induce luteinization of the granulosa cells, final oocyte maturation and resumption of meiosis. Since OHSS almost always presents either 3–7 days after hCG administration in susceptible patients (early onset) or during early pregnancy, 12–17 days after hCG administration (late onset), withholding the ovulation-inducing trigger of hCG with the consequent of cycle cancellation, was used to *eliminate* severe early OHSS. However, since cancellation denotes patient’s frustration and is associated with time and money consuming, other methods aiming to prevent OHSS while maintaining reasonable IVF outcome were suggested.

In 2000, Itskovitz-Eldor et al. [1] described the first series of patients, at risk to develop severe OHSS, that underwent COH using the GnRH- antagonist with GnRH-agonist (GnRHa) trigger for final follicular maturation. While 50 % conceived, none of the patients developed any signs or symptoms of OHSS. Controlled ovarian hyperstimulation (COH) which combines GnRH antagonist co-treatment and GnRHa trigger has since become a common tool aiming to *eliminate* severe early OHSS and to support the concept of an OHSS-free clinic [2, 3]. However, due to the reported significantly reduced clinical pregnancy and increased first trimester pregnancy loss [4, 5], efforts have been made to improve reproductive outcome by manipulating the luteal phase. One of the suggested optional strategies aiming to improve outcome was the addition of low-dose (1500 IU) HCG bolus.

## GnRHa and hCG in patients at risk to develop severe OHSS (Fig. 1)

**Fig. 1:**
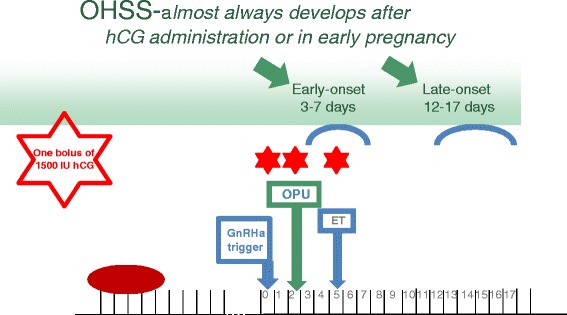
GnRHa and hCG trigger in patients at risk to develop severe OHSS

*One bolus of 1500 IU hCG* 35 h after the triggering bolus of GnRHa, i.e. one hour after oocyte retrieval [6, 7], was demonstrated to rescue the luteal phase, resulting in a reproductive outcome comparable with that of HCG triggering, and with no increased risk of OHSS [8]. However, when applied to patients at high-risk to develop severe OHSS, 26 % developed severe early OHSS requiring ascites drainage and hospitalization [9]. A figure that is comparable to the acceptable 20 % prevalence of severe OHSS in ostensibly high risk patients [10].

*One bolus of 1500 IU hCG* concomitant with GnRHa (dual trigger), 34–36 h before oocyte retrieval was suggested as a method which improves oocyte maturation, while providing more sustained support for the corpus luteum than can be realized by the GnRHa-induced LH surge alone [11, 12]. However, while acceptable rates of fertilization, implantation, clinical pregnancy, ongoing pregnancy rates, and early pregnancy loss were achieved in high responders after dual trigger [11, 12], the incidence of clinically significant OHSS was not eliminated, but rather reduced to 0.5 % [12].

*One bolus of 1500 IU hCG* five days after the triggering bolus of GnRHa [13, 14]. While the freeze-all policy was applied to all patients yielding more than 20 oocytes, those triggered with GnRHa, who achieved less than 20 oocytes, were instructed to start an intensive luteal support with estradiol and progesterone, the day following OPU, and were re-evaluated 3 days after oocyte retrieval (on day of embryo transfer) for signs of *early* moderate OHSS (ultrasonographic signs of ascites as reflected by the appearance of fluid surrounding the uterus/ovaries, and/or Hct levels >40 % for the degree of haemoconcentration). If no early signs of OHSS developed, one embryo was transferred, and the patients were instructed to inject 1500 IU of HCG. By deferring the hCG bolus by 3 days (5 days following GnRHa trigger), the corpus luteum was rescued, with an observed extremely high midluteal progesterone levels [14], reasonable pregnancy rate, with no patient developing severe OHSS. However, while these preliminary results are promising, the small sample size mandates further large prospective randomized studies [14].

## GnRHa versus hCG trigger- the physiological perspectives

In the course of the ovulatory cycle, sufficient production of estradiol by the preovulatory follicle induces the mid cycle LH surge, which is followed by a loss of gap junctions between the oocyte and cumulus cells, cumulus expansion, germinal vesicle breakdown, resumption of meiosis and luteinization of the granulosa cells. Moreover, the consequent increase in progesterone synthesis facilitates the positive feedback action of estradiol to induce the concomitant midcycle FSH peak [15]. This peak FSH has several roles, including the assurance of an adequate complement of LH receptors on the granulosa layer and the synthesis of hyaluronic acid matrix that facilitates the expansion and dispersion of the cumulus cells, allowing the oocyte-cumulus cell mass to become free-floating in the antral fluid [15].

As part of a standard/conventional COH regimen, final oocyte maturation and resumption of meiosis are usually triggered by one bolus of hCG (5000–10,000 units), that is administered as close as possible to the time of ovulation (i.e. 36 h before oocyte recovery [16]. In 1990, Gonen et al. [17] have demonstrated that ovulation may be also triggered by GnRHa, causing the release of both endogenous LH and FSH, mimicking the natural cycle surge and therefore considered to be more physiologic.

As a consequence of the aforementioned observations, several studies have emerged, comparing the effect of hCG versus GnRHa trigger on the different follicular maturation variables following an IVF treatment cycle. The number of oocytes retrieved, percentage of mature oocytes and number of top-quality embryos were either comparable or in favor of the GnRHa trigger (Table 1) [18–22] and might be explained by the following observations:

1. Unlike the GnRHa-induced mid-cycle surge of LH and FSH, terminating 24 h after its onset, the HCG-mediated LH activity, with no FSH rise, and spans several days into the luteal phase [18].
2. While both LH and hCG act on the same LH receptor, accumulating evidence suggests that LH has a greater impact on AKT and extracellular signal regulated protein kinase (ERK1/2) phosphorylation, responsible for granulosa cells proliferation, differentiation and survival, while hCG generates higher intracellular cAMP accumulation, which stimulates steroidogenesis (progesterone production) [23].

**Table 1:** The effect of GnRHa versus hCG trigger on the different follicular maturation variables following an IVF treatment cycle

Authors	#oocytes	#MII oocytes	#MII to #oocytes	#top quality embryos
Fauser et al. [18]	=		=	=
Kolibianakis et al. [19]	=	=	=	
Humaidan et al. [20]	=		>	
Acevedo et al. [21]	=	=	=	=
Erb et al. [22]	>	>		>

>In favor of GnRHa

## GnRHa and hCG in patients not at risk to develop severe OHSS (Fig. 2)

**Fig. 2:**
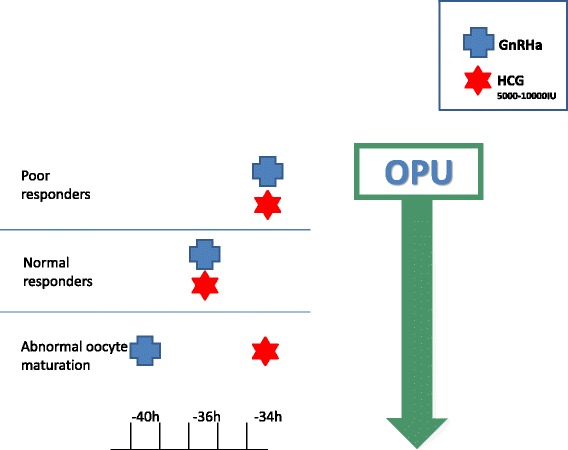
GnRHa and hCG trigger in patients not at risk to develop severe OHSS

The aforementioned observations demonstrating a comparable or even better oocyte\embryos maturity and quality following GnRHa trigger, as compared to hCG trigger, and the different effects of LH and hCG on the downstream signaling of the LH receptor, have led to a new strategy for final follicular maturation, the concomitant administration of both GnRHa and a standard bolus of hCG (5000–10,000 units) prior to oocyte retrieval, aiming to improve oocyte and embryo quality and the consequent IVF cycle outcome.

*Standard hCG dose* concomitant with GnRHa (dual trigger), 35–37 h before oocyte retrieval. Lin et al. [24], in their retrospective cohort study, have compared IVF outcome in normal responders patients undergoing COH using the GnRH antagonist with either a standard dosage of hCG trigger (6,500 IU of recombinant hCG) or the dual trigger (0.2 mg of triptorelin and 6,500 IU of recombinant hCG) 35–36 h prior to oocyte retrieval. The dual trigger group demonstrated statistically significantly higher number of oocytes retrieved, matured oocytes and number of embryos cryopreserved, with the consequent significant increase in implantation, clinical pregnancy and live-birth rates, as compared with the hCG-only trigger group.

In a subsequent prospective randomized controlled trial of normal responder patients, Decleer et al. [25] compared IVF outcome following either, 5000 IU of hCG trigger or a combination of GnRHa plus 5000 IU of hCG concomitantly, 36 h prior to oocyte retrieval. While no in between groups differences were observed in the mean number of oocytes retrieved, mature oocytes or pregnancy rates, the number of patients who received at least one embryo of excellent quality and the number of cryopreserved embryos were significantly higher following the dual trigger.

Griffin et al. [26] evaluated the effect of the dual trigger (GnRHa and hCG 5,000 IU or 10,000 IU, 35–37 h) prior to oocyte retrieval in patients with a previous history of >25 % immature oocytes retrieved. Despite a significantly higher proportion of mature oocytes retrieved with the dual trigger, the observed IVF outcome remained poor, probably due to patients’ underlying oocyte dysfunction.

*GnRHa 40 h and standard hCG* added 34 h prior to OPU (double trigger), respectively. Recently, Beck-Fruchter et al. [27] have described a case of recurrent empty follicle syndrome, successfully treated by ovulation trigger with GnRHa 40 h and hCG added 34 h prior to oocyte retrieval. They assumed that by prolonging the time between ovulation triggering and OPU [28] and the GnRHa trigger with the consequent simultaneous induction of an FSH surge, the “double trigger” could overcome any existing impairments in granulosa cell function, oocyte meiotic maturation or cumulus expansion, resulting in successful aspiration of mature oocytes, pregnancy and delivery.

Prompted by the aforementioned observations, we offered the double trigger to two group of patients demonstrating abnormal final follicular maturation despite normal response to COH, those with low (<50 %) number of oocytes retrieved per number of dominant follicles > 14 mm in diameter on day of hCG administration [29] and those with low proportion of mature/ metaphase-II (MII) oocytes (<66 %) per number oocytes retrieved [30].

In the group of patients with low (<50 %) number of oocytes retrieved per number of dominant follicles, following the double trigger, patients had significantly higher number of oocytes retrieved, number of 2PN, number of embryos transferred and significantly higher proportions of the number of oocytes retrieved to the number of follicles >10 mm and >14 mm in diameter on day of hCG administration, with a tendency toward a higher number of top-quality embryos, as compared to the hCG-only trigger cycles [29].

Moreover, in those with low proportion of MII oocytes (<66 %) per number oocytes retrieved, following the double trigger, patients yielded significantly higher number of MII oocytes and proportion of MII oocytes per number of oocytes retrieved, with the consequent significantly increased number of top-quality embryos, as compared to the hCG-only trigger cycles [30].

To conclude, when the effects of the dual and double triggers are observed across the aforementioned studies (Table 2), they are always in the same direction- consistently improved.

**Table 2:** The effect of Standard hCG dose concomitant with GnRHa (dual-double trigger) versus hCG alone on the different follicular maturation variables following an IVF treatment cycle

Authors	#oocytes	#MII oocytes	# embryos cryopreserved	#top quality embryos	Pregnancy rate
Lin et al. [24]	>	>	>	=	>
Decleer et al. [25]	=	=	=	=	=
			>patients with embryos	>patients with at least	
			Cryopreserved	one top quality embryo	
Griffin et al. [26]	>	>			=
Haas et al. [29]	>	>		>	>
Zilberberg et al. [30]	>	>		>	>

>In favor of the dual/double trigger

*Standard hCG dose* concomitant with GnRHa (dual trigger), 34 h before oocyte retrieval. According to the Bologna criteria, the minimal criteria needed to define poor ovarian respone (POR) are the presence of at least two of the following three features (i) Advanced maternal age (≥40 years) or any other risk factor for POR; (ii) A previous POR (≤3 oocytes with a conventional stimulation protocol); and (iii) An abnormal ovarian reserve test [31]. One of the major unnoticed concern in this group of poor responders is the observed high prevalence of premature luteinization\ovulation [32, 33], which may be overcome by early triggering of final follicular maturation- while approaching a follicular size of 15–16 mm, and by shortening the duration between the trigger and OPU. However, since shortening the interval between hCG priming and oocyte retrieval may decrease the percentage of mature oocytes [28], an additional measure to improve the number of oocytes retrieved to the number of follicles >10 mm, and the proportion of mature oocytes should be implemented [29, 30]. One of the suggested measures, that should be further study, is whether dual trigger (hCG and GnRHa) administered 34 h prior to OPU will provide the desired improve results.

## Conclusions

In the present review we analyzed and discuss the hitherto published studies relating to the different mode of GnRHa combined with hCG trigger- for final follicular maturation, aiming to elucidate how to tailor each mode to its appropriate subgroup of patients.

One bolus of 1500 IU hCG, concomitant, 35 h or 5 days after the triggering bolus of GnRHa, were all demonstrated to rescue the luteal phase, resulting in improved reproductive outcome in patients at risk to develop severe OHSS, as compared to GnRHa trigger alone, with the questionable ability to *eliminate* severe OHSS.

Moreover, following the observations demonstrating a comparable or even better oocyte\embryos quality following GnRHa trigger as compared to hCG trigger, and the different effects of LH and hCG on the downstream signaling of the LH receptor, GnRHa is now offered concomitant to the standard hCG trigger dose, to improve oocyte/embryo yield and quality. GnRHa and hCG may be offered concomitantly, 34–37 h prior to oocyte retrieval (dual trigger) or 40 h and 34 h prior to oocyte retrieval, respectively (double trigger) in patients with abnormal final follicular maturation.

Further large prospective studies are needed to examine the role of dual/double triggers in the different groups of patients undergoing IVF, prior to its routine implementation. Specifically, the ability of deferring the 1500 IU of hCG bolus to the day of embryo transfer to eliminate OHSS and the role of dual trigger 34 h prior to oocyte retrieval in poor responder patients, should be elucidated.

## Abbreviations

ART: Assisted reproduction technology
COH: Controlled ovarian hyperstimulation
GnRHa: GnRH agonist
hCG: Human chorionic gonadotropin
IVF: In vitro fertilization
OHSS: Ovarian hyperstimulation syndrome
